# Supplementary material for: The REGENERATE Study: A Non-Randomized Feasibility Study of an Intervention to REduce anticholinerGic burdEN in oldER pATiEnts
Source: Geriatrics (Basel). 2024 Nov 25;9(6):152. doi: 10.3390/geriatrics9060152 (PMC11728085; doi:10.3390/geriatrics9060152)
Supplement: Supplementary file 1 [file geriatrics-09-00152-s001.zip › geriatrics-3235277-supplementary.pdf]

Supplemental material S1 ACB intervention logic model

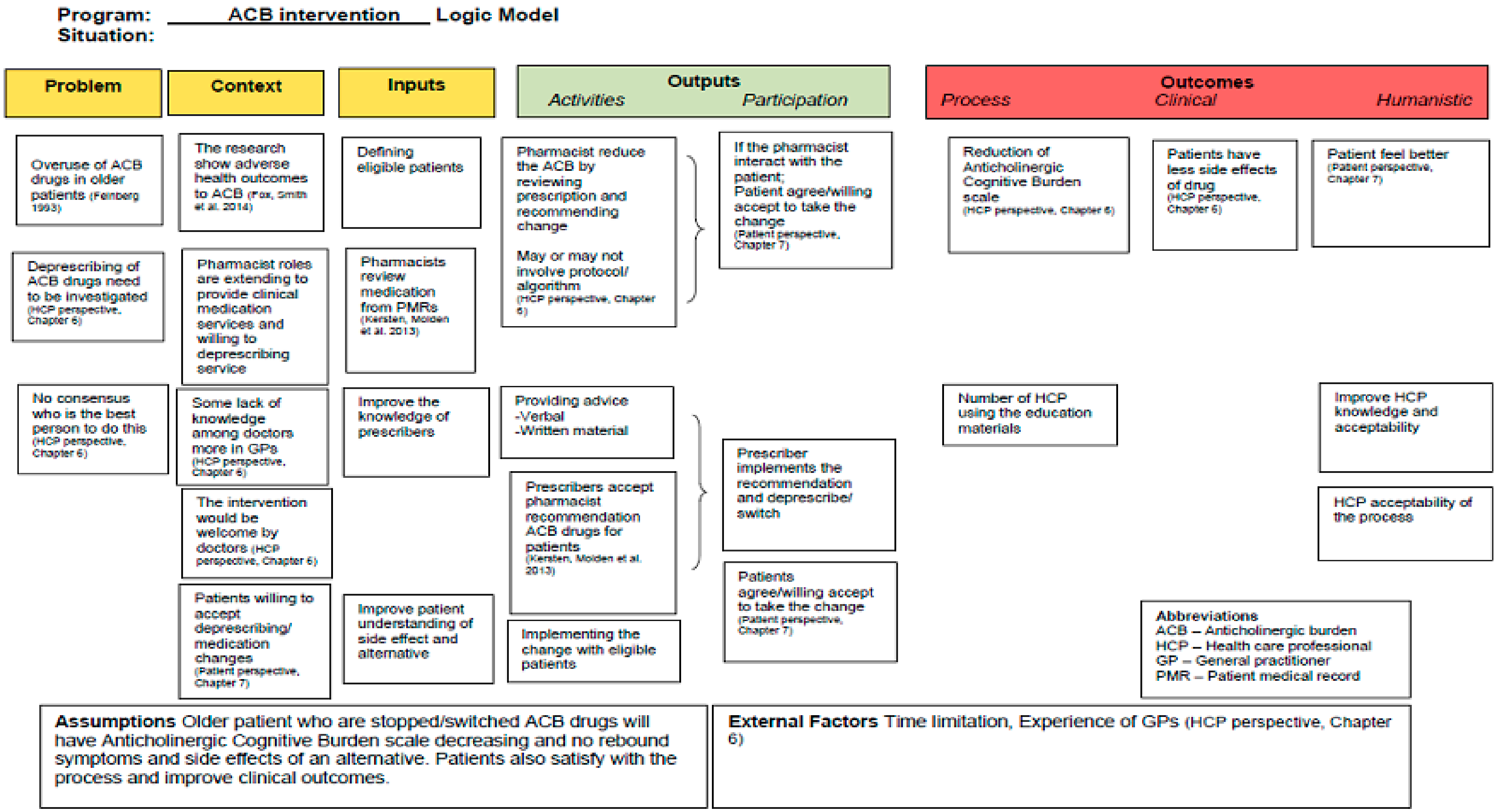

Supplemental material S2 Baseline, 6- and 12-weeks questionnaires for patient participants

Baseline questionnaire

Patient Questionnaire

REGENERATE STUDY

Dear Participant

Thank you for agreeing to take part in the REGENERATE study in which we are testing a new way of checking if there is a need to change any of the medicines you take. It is now important that we understand your experiences of taking part in the study to enable us to make improvements as we progress to the next stages of our research.

To collect that information, we are requesting you to complete the attached short questionnaire, and place the completed questionnaire into questionnaire return box in the surgery/clinic. Alternatively, you can take the questionnaire home, complete it and then please return it to us in the reply paid envelope that you can get from surgery/clinic staff. We will ask you to complete further similar questionnaires after 6 weeks and 3 months to collect all information required to assess the study results. Thank you again for your help with this study.

Yours sincerely,

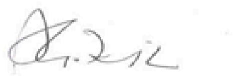

Professor Phyo Kyaw Myint
Chief Investigator

Please turn over

Patient ID..............

Patient questionnaire (Baseline)

Please respond to each of the following questions by ticking (✓) the relevant box or adding your comments where requested

Section 1: About you

What is your age? ☐ 65-69 ☐ 70-74 ☐ 75-79 ☐ 80-85 ☐ > 85

Are you? ☐ Male ☐ Female ☐ prefer not to say

Section 2: About your consultation with the pharmacist

1. Approximately how long was your first consultation with the pharmacist?
   ☐☐ Hours ☐☐ Minutes
2. Do you think the time was.... *(please tick one box only)*
   ☐ Too short? ☐ About right? ☐ Too long?
3. Was there anything you particularly liked about this first consultation with the pharmacist?

Please tell us about it briefly here

4. What (if anything) would encourage you to continue this service?

Please tell us about it briefly here

Please turn over

5. Was there anything you did not like about your consultation with the pharmacist?

Please tell us about it briefly here

6. Would this mean you did not want to use the service again?

Please tell us about it briefly here

Section 3-About the new approach to your medication

Please indicate your level of agreement with the following statements by placing a CROSS [X] in the appropriate box.

	Strongly agree	Agree	Uncertain/ not applicable	Disagree	Strongly disagree
1. The pharmacist appeared well informed.	☐	☐	☐	☐	☐
2. The pharmacist listened to what I had to say.	☐	☐	☐	☐	☐
3. The pharmacist answered all my concerns.	☐	☐	☐	☐	☐
4. I would rather have seen a doctor.	☐	☐	☐	☐	☐
5. I would rather have seen a nurse.	☐	☐	☐	☐	☐
6. I am completely happy with my consultation with the pharmacist	☐	☐	☐	☐	☐
7. It is a good idea to change my medication to reduce my chance of unwanted side effects	☐	☐	☐	☐	☐

Please turn over

Please add any other comments about the new approach to your medication that have not been covered by the questionnaire *(please state / describe below)*

Please turn over

Section 4 -About the study process

Please indicate your level of agreement with the following statements by placing a CROSS (X) in the appropriate box.

	Strongly agree	Agree	Uncertain/ not applicable	Disagree	Strongly disagree
1. The study purpose was clear.	☐	☐	☐	☐	☐
2. I was given enough information to allow me to decide whether to participate.	☐	☐	☐	☐	☐

Please add any other comments about research process that have not been covered by the questionnaire *(please state / describe below)*

....

....

....

....

....

....

....

....

....

Section 5- About your quality of life

Please complete the following questionnaire to assess your health and quality of life, by placing a CROSS (X) in the appropriate box.

Under each heading, please tick the ONE box that best describes your health TODAY

MOBILITY
- I have no problems in walking about ☐
- I have slight problems in walking about ☐
- I have moderate problems in walking about ☐
- I have severe problems in walking about ☐
- I am unable to walk about ☐

SELF-CARE
- I have no problems washing or dressing myself ☐
- I have slight problems washing or dressing myself ☐
- I have moderate problems washing or dressing myself ☐
- I have severe problems washing or dressing myself ☐
- I am unable to wash or dress myself ☐

USUAL ACTIVITIES *(e.g. work, study, housework, family or leisure activities)*
- I have no problems doing my usual activities ☐
- I have slight problems doing my usual activities ☐
- I have moderate problems doing my usual activities ☐
- I have severe problems doing my usual activities ☐
- I am unable to do my usual activities ☐

PAIN / DISCOMFORT
- I have no pain or discomfort ☐
- I have slight pain or discomfort ☐
- I have moderate pain or discomfort ☐
- I have severe pain or discomfort ☐
- I have extreme pain or discomfort ☐

ANXIETY / DEPRESSION
- I am not anxious or depressed ☐
- I am slightly anxious or depressed ☐
- I am moderately anxious or depressed ☐
- I am severely anxious or depressed ☐
- I am extremely anxious or depressed ☐

Part of EQ-5D from: EuroQol Research Foundation. EQ-5D-5L User Guide, 2019. Available from: https://euroqol.org/publications/user-guides.

Please add here any comments you have about the study

……………………………………………………………………………………………………

……………………………………………………………………………………………………

……………………………………………………………………………………………………

……………………………………………………………………………………………………

……………………………………………………………………………………………………

……………………………………………………………………………………………………

……………………………………………………………………………………………………

……………………………………………………………………………………………………

……………………………………………………………………………………………………

Thank you for taking the time to complete this questionnaire.

Please place the completed questionnaire in the return box in the surgery/clinic or prepaid envelope and send to:

<u>6- weeks questionnaire</u>

Dear Participant

Thank you for taking part in the REGENERATE study in which we are testing a new way of checking if there is a need to change any of the medicines you take. It is now important that we understand your experiences of taking part in this study. These will be used to inform our future work in this area.

To collect this information, we are inviting you to complete the attached questionnaire and return it to us in the reply paid envelope. We will be asking you to complete one more questionnaire at the end of the study, after approximately another 6 weeks.

Thank you once again for your help with this study

Yours sincerely,

Patient ID.....................

REGENERATE Patient questionnaire (6 weeks follow-up)

Please respond to each of the following questions by ticking (✓) the relevant box or adding your comments where requested

<u>**Section 1-About the new approach to your medication**</u>

Please indicate your level of agreement with the following statements by placing a tick (✓) in the appropriate box.

	Strongly agree	Agree	Uncertain/ not applicable	Disagree	Strongly disagree
1. I was happy to discuss my medicines with the pharmacist.	☐	☐	☐	☐	☐
2. The symptoms of my illness are controlled.	☐	☐	☐	☐	☐
3. I have no concerns related to their new approach for reviewing my medicines.	☐	☐	☐	☐	☐
**Please complete the following only if the pharmacists made changes to your medicines.**					
4. I am happy with the changes made to my medicines	☐	☐	☐	☐	☐
5. I am unhappy with the changes made to my medicines.	☐	☐	☐	☐	☐
6. I am currently on a new medication.	☐	☐	☐	☐	☐
7. I have changed back to my old medicines.	☐	☐	☐	☐	☐

Please explain if you were not satisfied about the medication changes. Have you contacted your prescriber about this issue?

Note: The patient information sheet contains contact number if you have any concern.

Have you had further contact with pharmacist, GP or nurse since your consultation with the pharmacist as part of this study? If yes, please describe these briefly in the box below, or write not applicable.

If have concerns about the changes to made to your medicines, please describe them briefly in the box below.

Please add any other comments about the new approach to your medication that have not been covered by the questionnaire items above *(please state / describe below)*

Section 2- About your quality of life

Please complete the following sections of this questionnaire, which will help us understand your health and quality of life, by placing a tick (✓) in the appropriate box.

Under each heading, please tick the ONE box that best describes your health TODAY

MOBILITY
I have no problems in walking about ❑
I have slight problems in walking about ❑
I have moderate problems in walking about ❑
I have severe problems in walking about ❑
I am unable to walk about ❑

SELF-CARE
I have no problems washing or dressing myself ❑
I have slight problems washing or dressing myself ❑
I have moderate problems washing or dressing myself ❑
I have severe problems washing or dressing myself ❑
I am unable to wash or dress myself ❑

USUAL ACTIVITIES *(e.g. work, study, housework, family or leisure activities)*
I have no problems doing my usual activities ❑
I have slight problems doing my usual activities ❑
I have moderate problems doing my usual activities ❑
I have severe problems doing my usual activities ❑
I am unable to do my usual activities ❑

PAIN / DISCOMFORT
I have no pain or discomfort ❑
I have slight pain or discomfort ❑
I have moderate pain or discomfort ❑
I have severe pain or discomfort ❑
I have extreme pain or discomfort ❑

ANXIETY / DEPRESSION
I am not anxious or depressed ❑
I am slightly anxious or depressed ❑
I am moderately anxious or depressed ❑
I am severely anxious or depressed ❑
I am extremely anxious or depressed ❑

Part of EQ-5D from: EuroQol Research Foundation. EQ-5D-5L User Guide, 2019. Available from: https://euroqol.org/publications/user-guides

Please add here any comments you have about the study.

……

……

……

……

……

……

……

……

……

Thank you for taking the time to complete this questionnaire.

Please return the questionnaire in the reply paid envelope and send to:

12- weeks questionnaire

Patient Questionnaire

REGENERATE STUDY

Dear Participant

Thank you for taking part in the REGENERATE study in which we are testing a new way of checking if there is a need to change any of the medicines you take. It is now important that we understand your experiences of taking part in this study. These will be used to inform our future work in this area.

To collect this information, we are inviting you to complete the attached questionnaire and return it to us in the reply paid envelope. We will be asking you to complete one more questionnaire at the end of the study, after approximately another 6 weeks.

Thank you once again for your help with this study

Yours sincerely,

Patient ID..............

REGENERATE Patient questionnaire (6 weeks follow-up)

Please respond to each of the following questions by ticking (✓) the relevant box or adding your comments where requested

Section 1-About the new approach to your medication

Please indicate your level of agreement with the following statements by placing a tick (✓) in the appropriate box.

	Strongly agree	Agree	Uncertain/ not applicable	Disagree	Strongly disagree
1. I was happy to discuss my medicines with the pharmacist.	☐	☐	☐	☐	☐
2. The symptoms of my illness are controlled.	☐	☐	☐	☐	☐
3. I have no concerns related to their new approach for reviewing my medicines.	☐	☐	☐	☐	☐
**Please complete the following only if the pharmacists made changes to your medicines.**					
4. I am happy with the changes made to my medicines	☐	☐	☐	☐	☐
5. I am unhappy with the changes made to my medicines.	☐	☐	☐	☐	☐
6. I am currently on a new medication.	☐	☐	☐	☐	☐
7. I have changed back to my old medicines.	☐	☐	☐	☐	☐

Please turn over

Please explain if you were not satisfied about the medication changes. Have you contacted your prescriber about this issue?

Note: The patient information sheet contains contact number if you have any concern.

Have you had further contact with pharmacist, GP or nurse since your consultation with the pharmacist as part of this study? If yes, please describe these briefly in the box below, or write not applicable.

If have concerns about the changes to made to your medicines, please describe them briefly in the box below.

Please add any other comments about the new approach to your medication that have not been covered by the questionnaire items above *(please state / describe below)*

Please turn over

<u>**Section 2- About your quality of life**</u>

Please complete the following sections of this questionnaire, which will help us understand your health and quality of life, by placing a tick (✓) in the appropriate box.

Under each heading, please tick the ONE box that best describes your health TODAY

MOBILITY
- **I have no problems in walking about** ❑
- **I have slight problems in walking about** ❑
- **I have moderate problems in walking about** ❑
- **I have severe problems in walking about** ❑
- **I am unable to walk about** ❑

SELF-CARE
- **I have no problems washing or dressing myself** ❑
- **I have slight problems washing or dressing myself** ❑
- **I have moderate problems washing or dressing myself** ❑
- **I have severe problems washing or dressing myself** ❑
- **I am unable to wash or dress myself** ❑

USUAL ACTIVITIES ***(e.g. work, study, housework, family or leisure activities)***
- **I have no problems doing my usual activities** ❑
- **I have slight problems doing my usual activities** ❑
- **I have moderate problems doing my usual activities** ❑
- **I have severe problems doing my usual activities** ❑
- **I am unable to do my usual activities** ❑

PAIN / DISCOMFORT
- **I have no pain or discomfort** ❑
- **I have slight pain or discomfort** ❑
- **I have moderate pain or discomfort** ❑
- **I have severe pain or discomfort** ❑
- **I have extreme pain or discomfort** ❑

ANXIETY / DEPRESSION
- **I am not anxious or depressed** ❑
- **I am slightly anxious or depressed** ❑
- **I am moderately anxious or depressed** ❑
- **I am severely anxious or depressed** ❑
- **I am extremely anxious or depressed** ❑

Part of EQ-5D from: EuroQol Research Foundation. EQ-5D-5L User Guide, 2019. Available from: https://euroqol.org/publications/user-guides

Please turn over

Please add here any comments you have about the study.

....

....

....

....

....

....

....

....

....

Thank you for taking the time to complete this questionnaire.

Please return the questionnaire in the reply paid envelope and send to:

Supplemental material S3 Interviews guides for doctors, pharmacists and patients.

Intervention to REduce anticholinerGic burdEN in oldER pATiEnts (REGENERATE) aged 65 years and older: A non-randomised feasibility study

Topic guide for doctors

INTRODUCTION

I'd like to thank you for agreeing to take part in this interview in which we want to get your feedback about taking part in the REGENERATE study.

As we explained in the information we sent you, we would like to record this interview. Is that OK?

SWITCH ON RECORDER

(Read out the participant ID for the purpose of recording and then take consent)

For our records please could you now verbally consent to us recording this interview?

- **Take informed consent from participants**
  (If interview is done by phone/video call, researcher will read through each point in the consent form and ask them to verbally agree to each point then audio recorded)
- **Re-iterate that the discussion will remain confidential and you will remain anonymous.**

Thank you

I now would like to discuss your experiences of taking part in the REGENERATE study. Firstly………

1. Can you tell me about your general experience of taking part in the study?
   - Prompt: What went well and what did not go so well?
   - Prompt: Any suggestions on what could be improved in a future study?

2. Thinking about delivering the intervention. Was there anything that made it easier or helped you to deliver the intervention? And anything that made it more difficult?

3. Overall, do you want to tell me anything more about,
   - delivering an ACB reduction intervention like this yourself
   - likelihood of changing their future prescribing decisions
   - receiving advice from pharmacists about ACB reduction (i.e. advising stopping/switching)?

4. The pharmacists in the study were given handouts on ACB reduction to use when talking party in the study. Were you aware of these?
   - Prompt: Were they useful?
   - Prompt: Were they used a lot?

5. As you know, this has been a feasibility study to inform a future randomised controlled trial. Would you be willing to take part in such a trial?

6. Would you have any recommendation for us about any changes we should make before finalising the main trial design?

I'd just like to take this opportunity to thank you again for coming today and taking part in this interview.

Intervention to REduce anticholinerGic burdEN in oldER pATiEnts (REGENERATE) aged 65 years and older: A non-randomised feasibility study

Topic guide for pharmacists

INTRODUCTION

I'd like to thank you for agreeing to take part in this interview in which we want to get your feedback about taking part in the REGENERATE study.

As we explained in the information we sent you, we would like to record this interview. Is that OK?

SWITCH ON RECORDER

(Read out the participant ID for the purpose of recording and then take consent)

For our records please could you now verbally consent to us recording this interview?

- Take informed consent from participants
  (If interview is done by phone/video call, researcher will read through each point in the consent form and ask them to verbally agree to each point then audio recorded)
- Re-iterate that the discussion will remain confidential and you will remain anonymous.

Thank you

I now would like to discuss your experiences of taking part in the REGENERATE study. Firstly……

1. Can you tell me about your general experience of taking part in the study?
   - Prompt: What went well and what did not go so well?
   - Prompt: Any suggestions on what could be improved in a future study?
2. Thinking about the training session that you attended as part of the research. Can you tell me how useful you have found it?
   - Prompt: Did you get new knowledge? Was it relevant to intervention? What would you change?

3. Now thinking a bit more about the training in relation to actually delivering the intervention. Did the training give you confidence? What about your knowledge and skills? Did you feel competent? Was there anything else you needed?
4. Was there anything that made it easier or helped you to deliver the intervention? And anything that made it more difficult?
5. Overall, do you want to tell me anything more about,
   - delivering ACB intervention like this yourself
   - giving advice to doctors about ACB reduction (i.e. advising stopping/switching)?
6. As you know, this is feasibility study to inform a future randomised controlled trial. Would you be willing to take part in such a trial?
7. Would you have any recommendation for us about any changes we should make before finalising the main trial design?

I'd just like to take this opportunity to thank you again for coming today and taking part in this interview.

Intervention to REduce anticholinerGic burdEN in oldER pATiEnts (REGENERATE) aged 65 years and older: A non-randomised feasibility study

Topic guide for patients

INTRODUCTION

I'd like to thank you for agreeing to take part in this interview in which we want to get your feedback about taking part in the REGENERATE study.

As we explained in the information we sent you, we would like to record this interview. Is that OK?

SWITCH ON RECORDER

(Read out the participant ID for the purpose of recording and then take consent)

For our records please could you now verbally consent to us recording this interview?

- Take informed consent from participants
  (If interview is done by phone/video call, researcher will read through each point in the consent form and ask them to verbally agree to each point then audio recorded)
- Re-iterate that the discussion will remain confidential and you will remain anonymous.

Thank you

I now would like to discuss your experiences of taking part in the REGENERATE study. Firstly……

1. Firstly, I want to check you remember taking part in the study? Can you tell me what it was about and your general experience?
    - Prompt: check they knew what it was all about and its purpose
    - Prompt: What went well and what did not go so well?
    - Prompt: Any suggestions on what could be improved in a future study?

2. Did you have any difficulties taking part in REGENERATE study? What are they? Any suggestions how we could improve this in the future?
    - Prompt: Did you have any concerns when the prescribing changed
    - Prompt: Any worries?
    - Prompt: Any change in symptoms?

3. If we were to conduct a bigger study in the future, would you be willing to take part?
    - Prompt: If yes (say thanks) and ask to explain
- Prompt: If no ask to explain

4. Is there anything else that I have not covered but you would like to discuss?

   Highlight again that everything we discussed today will remain confidential

I'd just like to take this opportunity to thank you again for coming today and taking part in this interview.

Author Contributions: All authors have substantial contributions to the conception or design of the work, analysis, or interpretation of data for the work, AN drafted the work and other authors revised paper critically for important intellectual content, final approval of the version to be published and agreement to be accountable for all aspects of the work in ensuring that questions related to the accuracy or integrity of any part of the work are appropriately investigated and resolved.

Conceptualization, A.N, C.B., M.C, R.N. and P.K.M.; Methodology, A.N, C.B., M.C, R.N. and P.K.M.; Investigation, A.N.; Data Curation, A.N. and P.K.M.; Writing – Original Draft Preparation, A.N.; Writing – Review & Editing, C.B., M.C, R.N. and P.K.M.

Funding: This research was funded by the University of Aberdeen.

Institutional Review Board Statement: The study was conducted according to the guidelines of the Declaration of Helsinki and approved by the East of Scotland Research Ethics Committee (protocol code: 2-094-20 and date of approval: 8 April 2021).

Informed Consent Statement: Informed consent was obtained from all subjects involved in the study.

Acknowledgments: We would like to thank Mrs. Valerie Sillito for her role as a primary care pharmacist in this study.

Conflicts of Interest: The authors declare no conflict of interest.

References:

1. World Health Organization, 2021-last update, Ageing and health. Available: https://www.who.int/news-room/fact-sheets/detail/ageing-and-health [12/August, 2021].
2. Maher, R.L., Hanlon, J.T. and Hajjar, E.R., 2014. Clinical consequences of polypharmacy in elderly. *Expert Opinion on Drug Safety,* 2014, 13(1), pp. 1-11.
3. Katzenschlager, R., Sampaio, C., Costa, J. and Lees, A. Anticholinergics for symptomatic management of Parkinson´s disease. *Cochrane Database of Systematic Reviews,* 2022, (3), pp. 1-19.
4. O'Donnell, K.L., Gnjidic, D., Nahas, R., Bell, J.S. and Hilmer, S.N. Anticholinergic burden: Considerations for older adults. *Journal of Pharmacy Practice and Research,* 2017 47(1), pp. 67-77.
5. Vardanyan, R.S. and Hruby, V.J. Anticholinergic drugs. In: R.S. VARDANYAN and V.J. HRUBY, eds, *Synthesis of essential drugs,* 2006. Elsevier, pp. 195-208.
6. Rudolph, J.L., Salow, M.J., Anglini, M.C. and Mcglinchey, R.E. The anticholinergic risk scale and anticholinergic adverse effects in older persons. *Arch Intern Med,* 2008, 168(5), pp. 508-513.
7. Ancelin, M.L., Artero, S., Portet, F., Dupuy, A., Touchon, J. and Ritchie, K. Non-degenerative mild cognitive impairment in elderly people and use of anticholinergic drugs: longitudinal cohort study. *British Medical Journal,* 2006, 332, pp. 455-459.

8. Ehrt, U., Broich, K. and Larsen, J.P. Use of drugs with anticholinergic effect and impact on cognition in Parkinson's disease: A cohort study. *Journal of Neurology, Neurosurgery, and Psychiatry,* 2010, 81, pp. 160-165.
9. Kersten, H. and Wyller, T.B. Anticholinergic drug burden in older people's brain - how well is it measured? *Basic & clinical pharmacology & toxicology,* 2014, 114(2), pp. 151-9.
10. Ablett, A.D., Wood, A.D., BarrA, R., Guillot, J., Black, A.J., Macdonald, H.M., Reid, D.M. and Myint, P.K. A high anticholinergic burden is associated with a history of falls in the previous year in middle-aged women: findings from the Aberdeen prospective osteoporosis screening study. *Annals of Epidemiology,* 2018, 28, pp. 557-562.
11. Nakham, A., Myint, P.K., Bond, C.M., Newlands, R., Loke, Y.K. and Cruickshank, M. Interventions to reduce anticholinergic burden in adults aged 65 and older: A systematic review. *Journal of the American Medical Directors Association,* 2020, 21(2), pp. 170-180.
12. Krishnaswami, A., Steinman A. M., Goyal, P., Zullo, R.A., Anderson, S.T., Birtcher, K.K., Goodlin, J.S., Maurer, S.M., Alexander, P.K., Rich, W.M. and Tjia, J. Deprescribing in older adults with cardiovascular disease. *Journal of the American College of Cardiology,* 2019, 73(20), pp. 2584-2595.
13. Michael, S. and Reeve, E. *Deprescribing.* https://www.uptodate.com/contents/deprescribing#:~:text=Common%20goals%20for%20deprescribing%20include,to%20improving%20quality%20of%20life. edn. UpToDate, 2022.
14. Olasehinde-Williams, O., July, 2020-last update, Deprescribing guide, 2020. Available: https://southendccg.nhs.uk/your-health-services/healthcare-professionals/medicines-management/medicines-management-resources/2308-deprescribing-guide/file [April/03, 2020].
15. Craig, P., Dieppe, P., Macintyre, S., Michie, S., Nazareth, I. and Petticrew, M., 2019-last update, Developing and evaluating complex interventions: Following considerable development in the field since 2006, MRC and NIHR have jointly commissioned an update of this guidance to be published in 2019. Available: https://mrc.ukri.org/documents/pdf/complex-interventions-guidance/ [05/01, 2021].
16. Stewart, C., Gallacher, K., Nakham, A., Cruickshank, M., Newlands, R., Bond, C., Myint, P.K., Bhattacharya, D. and Mair, F.S. Barriers and facilitators to reducing anticholinergic burden: a qualitative systematic review. *International Journal of Clinical Pharmacy,* 2021, 43, pp. 1451-1460.
17. Cunningham, Y., Wood, C., Stewart, C., Nakham, A., Newlands, R., Gallacher, K.I., Quinn, T.J., Ellis, G., Lowrie, R., Myint, P.K., Bond, C. and Mair, F.M. Understanding Stakeholder Views Regarding the Design of an Intervention Trial to Reduce Anticholinergic Burden: A Qualitative Study. *Frontiers in Pharmacology,* 2021, 12, pp. 1-10.
18. Boustani, M., Campbell, N., Munger, S., Maidment, I. and Fox, C. Impact of anticholinergics on the aging brain: A review and practical application. *Aging Health,* 2008, 4(3), pp. 311-320.
19. Bruhn, H., Bond, C.M., Elliott, A.M. Pharmacist led management of chronic pain in primary care: results from a randomised controlled exploratory trial. *BMJ Open*, 2013, 3:e002361. doi:10.1136/bmjopen-2012

20. Wickware, C. When will England get a 'Pharmacy First' service? *The Pharmaceutical Journal*, 2023, 310(7969): DOI: 10.1211/PJ.2022.1.126642
21. Okeowo, D.A., Zaidi, S.T.R., Fylan, B. and Alldred, D.P. Barriers and facilitators of implementing proactive deprescribing within primary care: a systematic review. *International Journal of Pharmacy Practice*, 2023, 31(2), pp.126-152.
22. Kua, C., Mak, V.S.L. and Lee, S.W.H. Health Outcomes of Deprescribing Interventions among older residents in nursing homes: A systematic review and meta-analysis. *Journal of the American Medical Directors Association,* 2019, 20(3), pp. 362-372.
23. Thillainadesan, J., Gnjidic, D., Green, S. and Hilmer, S.N. Impact of deprescribing interventions in older hospitalised patients on prescribing and clinical outcomes: a systematic review of randomised trials. *Drugs Aging,* 2018, 35(4), pp. 303-319.
24. Ulley, J., Harrop, D., Ali, Ali, Alton, S. and Davis, S.F. Deprescribing interventions and their impact on medication adherence in community-dwelling older adults with polypharmacy: a systematic review. *BMC Geriatrics,* 2019, 19(15), pp. 1-13.
